# Multicenter cohort-study of 15326 cases analyzing patient satisfaction and perioperative pain management: general, regional and combination anesthesia in knee arthroplasty

**DOI:** 10.1038/s41598-018-22146-7

**Published:** 2018-02-27

**Authors:** Felix Greimel, Guenther Maderbacher, Clemens Baier, Armin Keshmiri, Timo Schwarz, Florian Zeman, Winfried Meissner, Joachim Grifka, Achim Benditz

**Affiliations:** 1 Department of Orthopedics, University Medical Center Regensburg, Asklepios Klinikum Bad Abbach, Kaiser-Karl-V.-Allee 3, 93077 Bad Abbach, Germany; 2 Center for Clinical Studies, University Medical Center of Regensburg, Franz-Josef-Strauss-Allee 11, 93053 Regensburg, Germany; 3 Department of Anesthesiology and Intensive Care, Jena University Hospital, Am Klinikum 1, 07747 Jena, Germany

## Abstract

Numbers of knee replacement surgeries have been rising over the past years. After having ameliorated operation techniques and material, pain management and anesthetic methods have come into focus. All 15326 patients included had undergone primary knee arthroplasty within this multicenter cohort-study, conducted in 46 orthopedic departments. Parameters were evaluated on first postoperative day. Primary outcome values were pain levels (activity, minimum and maximum pain, and pain management satisfaction). Pain medication necessity was analyzed. Parameters were compared between the types of anesthesia used: general, regional and combination anesthesia. Pain scores and pain management satisfaction were significantly better in the groups of either spinal or peripheral anesthesia combined with general anesthesia (p < 0.001, respectively). Patients who received the combination of general and spinal anesthesia were associated with the lowest need for opioids (p < 0.001). The use of a combined general and spinal anesthesia as well as using a combination of general and peripheral anesthesia in knee arthroplasty was associated with a highly significant advantage to other anesthetic techniques regarding perioperative pain management in daily clinical practice, but maybe below clinical relevance. Furthermore they were associated with positive tendency considering side effects and subjective well-being parameters.

## Introduction

Numbers of knee replacement surgeries have been rising over the past years, especially due to demographic changes^1^. Although perioperative pain might lead to delayed mobilization and thereby impair the overall outcome after knee arthroplasty, postoperative pain management is still controversially discussed. In the past, a couple of trials favored peripheral or epidural anesthesia^2,3^.

Usually, pain medication used in the postoperative course comprises non-opioid drugs and opioids, both potentially causing side effects. Thus, the choice of anesthetic methods and its influence on the need of postoperative pain medication as well as its effect on patient-reported outcome parameters is of high interest. The risk of chronic pain after total knee replacement surgery has been reported to occur in up to 1/3 of the cases^4^ and high postoperative pain levels seem to raise the risk of chronic pain^5,6^. In addition, patients’ recovery and mobilization ability might be improved, which could potentially shorten length of hospital stay and reduce postoperative complications. This, after all, might also reduce costs on the medical system.

Beneath other factors, type of anesthesia might influence postoperative pain.

Most commonly used anesthesia methods throughout the operation for knee replacement surgery are general and spinal anesthesia as well as peripheral nerve blocks or the combination of the mentioned methods. In the past years, many studies have favored regional anesthesia over general anesthesia for various reasons. Regional anesthesia was found to cause less cardiovascular and pulmonary complications^3^, reduce mortality^7^, have less blood loss as well as a lower transfusion rates^8^, show smaller amounts of site infections^9^ and shorter duration of operation^10^. In conclusion, these factors allow faster rehabilitation and shorten the duration of inpatient treatment^3,7^ thus resulting in economization of the health care system^11,12^.

Peripheral nerve blocks (PNBs) are another regional anesthetic method used to control intraoperative pain, possibly combined with both general or spinal anesthesia. Peripheral nerve blocks have been shown to be able to reduce opioid consumption and lower cardiac, pulmonary and thromboembolic risk^13^.

Adverse effects of regional anesthesia such as urinary retention and lack of bladder control, perineural hematoma or neural infection seem to account for patients’ fear of regional anesthesia^14^. Both hematoma and infection may also occur after neural blocks. However, perineural hematoma seems to occur more likely after epidural anesthesia^15–17^. Neural infections may occur, but it still should be considered that the risk of infection is rather low compared to the risk of neural deficits caused by the surgery itself^18^.

In many hospitals, general anesthesia is still preferred to regional anesthesia^3^ because of lack of expertise by anesthesiologists and shortage of time^14^. The utilization rate of peripheral nerve blocks seems to be comparably low^19^. Thus, it can be stated that general anesthesia is still widely performed. The combination of general anesthesia and regional anesthesia has been investigated in the past and shown a decrease of blood loss compared to general anesthesia only^7,20^.

In the postoperative course the conventional treatment consists of non-opioid drugs plus, if necessary, opioids. The combination of Non-steroidal anti-inflammatory drugs (NSAID) and regional anesthesia has shown to be able to decrease postoperative pain scores^21,22^. To improve postoperative pain management and knowing the side effects of pain medication, finding the anesthetic method leading to least amounts of postoperative pain medication is essential. Optimal pain treatment demands sufficient staff education as well as multidisciplinary cooperation, which is often lacking^23^.

Insufficient pain management can be detected by continuous quality improvement (CQI) strategies. By using this knowledge, the “Quality Improvement in Postoperative Pain Management” (QUIPS) project is an outstanding tool to assess and compare pain management.

## Purpose

This large-scale multicenter study evaluates different types of anesthesia in knee replacement surgery in daily routine, in order to compare general, spinal and combined general and regional anesthesia regarding need for pain medication, subjective functional scores as well as pain scores and patient’s satisfaction in the postoperative course. We hypothesized that regional anesthesia and combined regional and general anesthesia is superior to general anesthesia in terms of postoperative pain values and pain medication use.

## Material and Methods

In the present cohort study 15326 patients were evaluated between 2009 and 2015 after primary knee arthroplasty, conducted nationwide in 46 orthopedic departments at the time of data evaluation. All knee replacement surgeries meeting the inclusion/exclusion criteria were evaluated. Inclusion criteria were: (1) patients older than 18 years of age (2) able to communicate adequately (3) after primary knee arthroplasty. Written and oral information was supplied to all potential patients. Participation was voluntarily. A written informed consent to participate was received by every subject. Exclusion criteria were: (1) patients who refused to participate and (2) patients who were asleep or sedated at the time of data collection and interview on first postoperative day, respectively.

Within the scope of the “Quality Improvement in Postoperative Pain Management” (QUIPS) project, an initiative to compare outcome parameters in participating hospitals, parameters were analyzed on 1^st^ postoperative day. The QUIPS-project is endorsed by the German Society of Surgeons and the German Society of Anesthesiologists^24,25^.

Using the standardized QUIPS questionnaire form, minimum, maximum and activity pain as well as pain management satisfaction was rated using the numerous rating scale (NRS: NRS 0 = no pain or very unsatisfied, NRS 10 = worst pain imaginable or highly satisfied). Furthermore, side effects were evaluated with the two possible answers “Yes” or “No”, and percentages of patients answering “Yes” was calculated: “Pain affecting the ability to sleep”, “Pain affecting the ability to move the operated leg”, “Vomiting since surgery”, “Pain affecting the ability to cough or to take a deep breath”, “Tired since surgery”, “Felt vertiginous since surgery”, “Pain affecting the mood” and “Felt nauseous since surgery”. Postoperative pain medication was obtained from patients’ records. Type of postoperative pain medication was classified according to the WHO pain ladder: non-opioids (WHO ladder step 1) and opioids (summarization of WHO ladder step 2 and step 3)^26^. The medication was registered as “received” or “not received”, and percentages of patients who received this type of pain medication were calculated. The use of patient-controlled analgesia was recorded as well. The use of PCA was registered as “received” or “not received” as mentioned above.

To avoid selection bias and patient-interviewer interaction bias, all patients were randomly visited and informed by the staff member that their work is independent from the healthcare team. The interviewers were blinded to the anesthetic technique used. Data was anonymized after collection.

Standardized data assessment in each site to collect clinical data and obtain the questionnaire parameters was guaranteed due to data collection by specially educated staff using a standardized protocol. A multidisciplinary team of anesthesiologists, orthopedic surgeons and nurses regularly analyses data for internal benchmarking and comparison to other departments.

Patients were divided into five groups: (1) general, (2) spinal, and the combination of (3) spinal and peripheral, (4) general and spinal and (5) general and peripheral anesthesia. The groups were defined as following: all general narcotic techniques were summarized in group 1, all neuroaxial blocks in group 2, all peripheral nerve blocks (PNB) in combination with neuroaxial blocks in group 3, all sedation techniques in combination with neuroaxial blocks in group 4 and all sedation techniques in combination with PNBs in group 5.

### Statistics

Continuous variables are presented as mean (standard deviation) or as median (interquartile range) depending on the underlying distribution. Categorical data are presented as absolute numbers and/or relative frequencies. To compare the need for pain medication, side effects and functional parameters between the study groups, a Pearson’s chi-squared test was used for each pairwise comparison. Differences in NRS values between the study groups were analyzed using an analysis of variance (ANOVA) with Bonferroni pairwise post-hoc tests. A p-value < 0.05 was considered statistically significant. No imputation methods were performed. All analyses were performed using SPSS 22.0 (IBM SPSS Statistics, Armonk, NY – IBM Corp.).

### Ethics information

The study was approved by the Ethics Committee as well as the Data Security Board of the Jena University Hospital, Jena, Germany, as well as by the Ethics Committee of the University of Regensburg. Furthermore, the project was registered in the German Register of Clinical Studies (DRKS) with the approval number DRKS00006153 (WHO register). The study was applied in accordance with the ethical standards of the Declaration of Helsinki 1975.

## Results

**Figure 1:**
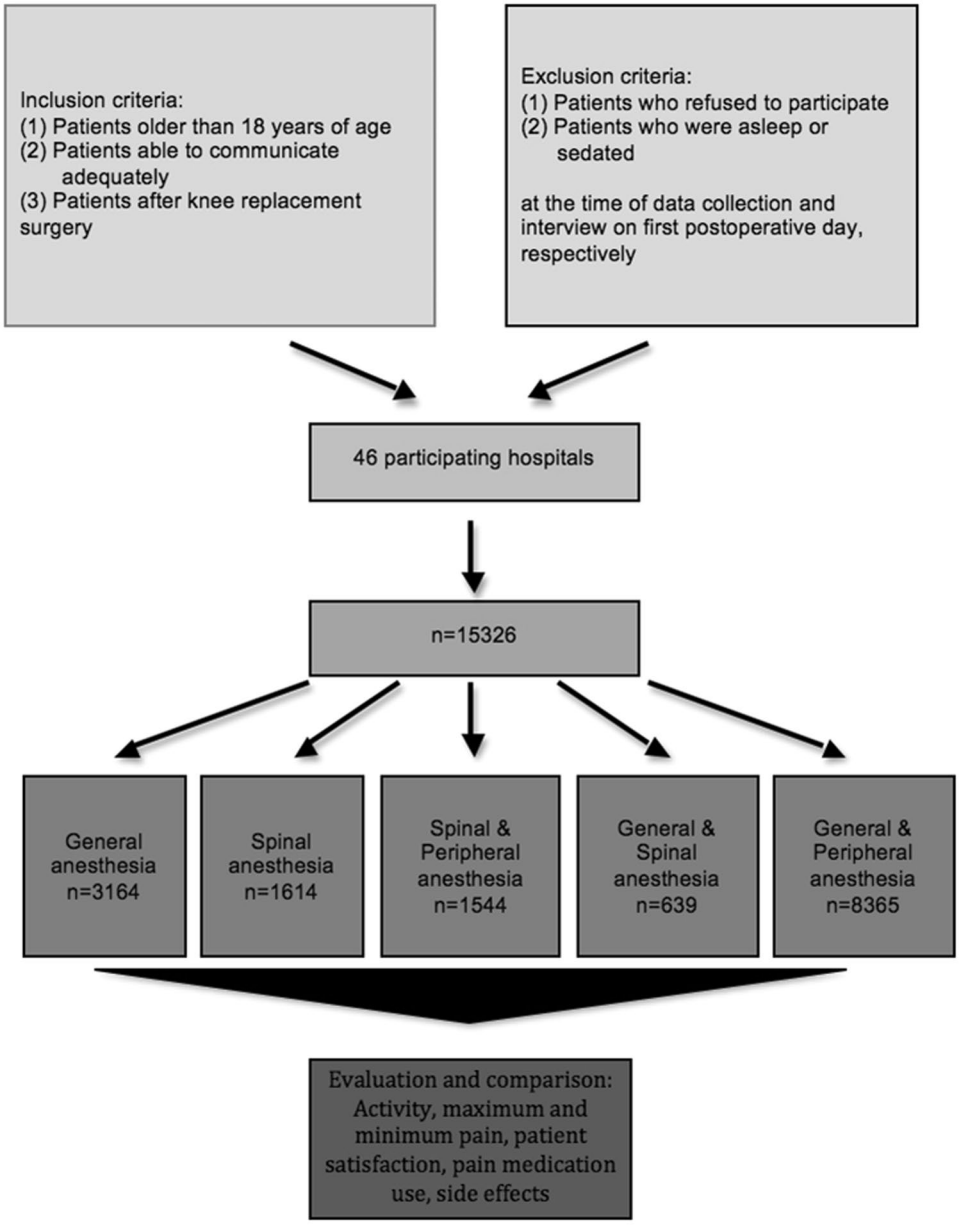
Flowchart: study group enrollment.

15326 patients were enrolled and evaluated after receiving knee arthroplasty between 2009 and 2015 in the present study (Fig. 1).

Mean age regarding all patients was 67.1 years (±10.2), 61.6% of all patients were female, the median American Society of Anesthesiologists (ASA) score was 2 (IQR 2–3). Mean duration of surgery was 89 minutes (±32) regarding all groups. Pain levels before surgery showed a median of 7 (IQR 2–8).

The groups in question, demographic and general data were homogenous (Table 1).

**Table 1:** Demographic and general data.

	General	Spinal	Spinal & Peripheral	General & Spinal	General & Peripheral
**Patients total** (%)	3164 (20.2)	1614 (10.3)	1544 (9.9)	639 (4.1)	8365 (53.4)
**Age in years** [Mean ± SD]	67.0 ± 10.4	68.5 ± 9.6	68.2 ± 10.0	66.6 ± 10.2	66.7 ± 10.2
**Sex in %** [female: male]	64.4: 35.6	58.7: 41.3	56.8: 43.2	58.2: 41.8	62.1: 37.9
**ASA score** [Median, Mean ± SD]	2, 2.2 ± 0.7	2, 2.3 ± 0.6	2, 2.4 ± 0.6	2, 2.3 ± 0.5	2, 2.2 ± 0.6
**Op time in min** [Median, Mean ± SD]	88, 93.0 ± 30.9	90, 94.5 ± 30.8	83, 85.3 ± 26.5	74, 81.9 ± 33.8	82, 87.7 ± 32.5
**Pain before surgery NRS** [Median, Mean ± SD]	7, 6.6 ± 2.0	7, 6.6 ± 2.0	7, 6.6 ± 2.0	6, 6.3 ± 2.0	7, 6.5 ± 2.0

**Figure 2:**
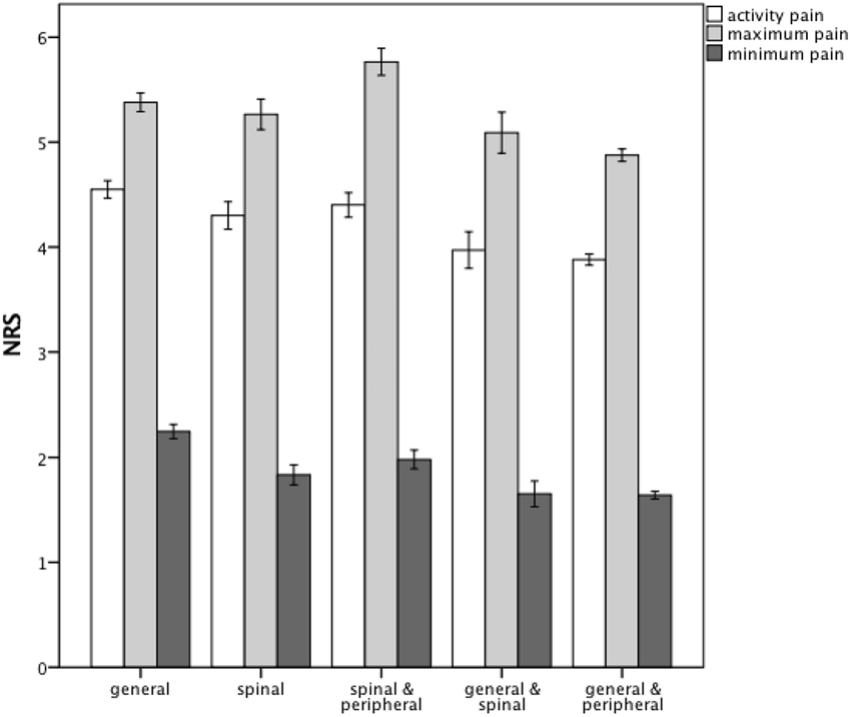
Barcharts: mean NRS values and 95%-confidence intervals for activity pain, maximum pain and minimum pain - grouped by anesthetic techniques on 1st postoperative day.

Mean activity pain was 4.6 (±2.3) in the general anesthesia group, 4.3 (±2.7) in the spinal anesthesia group, 4.4 (±2.3) in the combination of spinal and peripheral anesthesia group, 4.0 (±2.2) in the combination of general and spinal anesthesia group and 3.9 (±2.5) in the combination of general and peripheral anesthesia group. (Fig. 2) Significance levels are shown in Table 2.

**Table 2:** Comparison of activity pain, maximum pain, minimum pain and pain management satisfaction between the different anesthetic groups: mean values, standard deviation and their significance levels.

		Spinal		Spinal & Peripheral		General & Spinal		General & Peripheral	
		Activity pain	4.3 ± 2.7	Activity pain	4.4 ± 2.3	Activity pain	4.0 ± 2.2	Activity pain	3.9 ± 2.5
		Maximum pain	5.3 ± 2.9	Maximum pain	5.8 ± 2.6	Maximum pain	5.1 ± 2.5	Maximum pain	4.9 ± 2.7
		Minimum pain	1.8 ± 1.9	Minimum pain	2.0 ± 1.8	Minimum pain	1.7 ± 1.6	Minimum pain	1.6 ± 1.7
		Pain management satisfaction	8.0 ± 1.9	Pain management satisfaction	8.0 ± 2.0	Pain management satisfaction	8.1 ± 1.8	Pain management satisfaction	8.4 ± 1.8
**General**									
Activity pain	4.6 ± 2.4	p = 0.012		p = 0.655		p < 0.001		p < 0.001	
Maximum pain	5.4 ± 2.4	p = 1.0		p < 0.001		p = 0.169		p < 0.001	
Minimum pain	2.3 ± 1.9	p < 0.001		p < 0.001		p < 0.001		p < 0.001	
Pain management satisfaction	8.0 ± 1.9	p = 1.0		p = 1.0		p = 1.0		p < 0.001	
**Spinal**									
Activity pain	4.3 ± 2.7			p = 1.0		p = 0.063		p < 0.001	
Maximum pain	5.3 ± 2.9			p < 0.001		p = 1.0		p < 0.001	
Minimum pain	1.8 ± 1.9			P = 0.413		p = 0.389		p = 0.001	
Pain management satisfaction	8.0 ± 1.9			p = 1.0		p = 1.0		p < 0.001	
**Spinal & Peripheral**									
Activity pain	4.4 ± 2.3					p = 0.003		p < 0.001	
Maximum pain	5.8 ± 2.6					p < 0.001		p < 0.001	
Minimum pain	2.0 ± 1.8					p = 0.002		p < 0.001	
Pain management satisfaction	8.0 ± 2.0					p = 0.463		p < 0.001	
**General & Spinal**									
Activity pain	4.0 ± 2.2							p = 1.0	
Maximum pain	5.1 ± 2.5							p = 1.0	
Minimum pain	1.7 ± 1.6							p = 1.0	
Pain management satisfaction	8.1 ± 1.8							p = 0.007	

Mean maximum pain was 5.4 (±2.5) in the general anesthesia group, 5.3 (±2.9) in the spinal anesthesia group, 5.8 (±2.6) in the combination of spinal and peripheral anesthesia group, 5.1 (±2.5) in the combination of general and spinal anesthesia group and 4.9 (±2.7) in the combination of general and peripheral anesthesia group. (Fig. 2). Significance levels are shown in Table 2.

Mean minimum pain was 2.3 (±1.9) in the general anesthesia group, 1.8 (±1.9) in the spinal anesthesia group, 2.0 (±1.8) in the combination of spinal and peripheral anesthesia group, 1.7 (±1.6) in the combination of general and spinal anesthesia group and 1.6 (±1.7) in the combination of general and peripheral anesthesia group. (Fig. 2). Significance levels are shown in Table 2.

**Figure 3:**
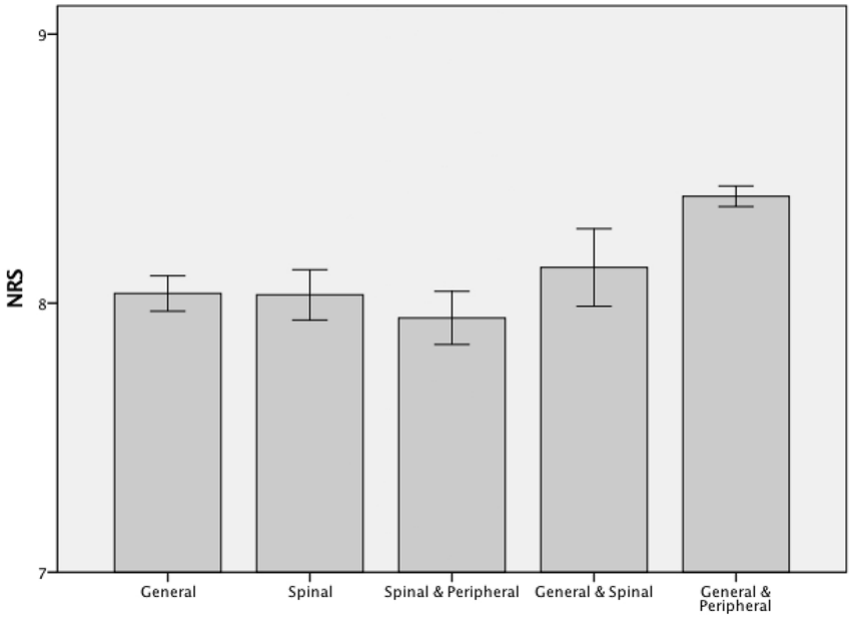
Barcharts: mean NRS values and 95%-confidence intervals for pain management satisfaction - grouped by anesthetic techniques on 1st postoperative day.

Mean pain management satisfaction was 8.0 (±1.9) in the general anesthesia group, 8.0 (±1.9) in the spinal anesthesia group, 8.0 (±2.0) in the combination of spinal and peripheral anesthesia group, 8.1 (±1.8) in the combination of general and spinal anesthesia group and 8.4 (±1.8) in the combination of general and peripheral anesthesia group. (Fig. 3). Significance levels are shown in Table 2.

The postoperative need for opioid pain medication and their significance levels are shown in Table 3.

**Table 3:** Comparison of the need for pain medication until 1^st^ postoperative day between the different anesthetic groups: mean values, standard deviation and their significance levels.

		Spinal		Spinal & Peripheral		General & Spinal		General & Peripheral	
		Non-opioids	86.1%	Non-opioids	95.2%	Non-opioids	96.1%	Non-opioids	93.4%
		Opioids	73.2%	Opioids	74.6%	Opioids	36.1%	Opioids	67.8%
		PCA	81.7%	PCA	16.7%	PCA	89.5%	PCA	9.8%
**General**									
Non-opioids	93.7%	p < 0.001		p = 0.043		p = 0.024		p = 0.596	
Opioids	74.3%	p = 0.433		p = 0.830		p < 0.001		p < 0.001	
PCA	77.3%	p < 0.001		p < 0.001		p < 0.001		p < 0.001	
**Spinal**									
Non-opioids	86.1%			p < 0.001		p < 0.001		p < 0.001	
Opioids	73.2%			p = 0.386		p < 0.001		p < 0.001	
PCA	81.7%			p < 0.001		p < 0.001		p < 0.001	
**Spinal & Peripheral**									
Non-opioids	95.2%					p = 0.398		p = 0.010	
Opioids	74.6%					p < 0.001		p < 0.001	
PCA	16.7%					p < 0.001		p = 0.009	
**General & Spinal**									
Non-opioids	96.1%							p = 0.010	
Opioids	36.1%							p < 0.001	
PCA	89.5%							p < 0.001	

Side effects “Pain affecting the ability to sleep”, “Pain affecting the ability to move”, “Vomiting since surgery”, “Pain affecting the ability to cough or to take a deep breath”, “Tired since surgery”, “Felt vertiginous since surgery”, “Pain affecting the mood” and “Felt nauseous since surgery”, depending on the anesthetic technique used intraoperatively are shown in Table 4.

**Table 4:** Side effects and subjective parameters on 1^st^ postoperative day.

	General	Spinal	Spinal & Peripheral	General & Spinal	General & Peripheral
Pain affecting the ability to sleep	45.1%	45.0%	48.1%	53.5%	39.0%
Pain affecting the ability to move the operated leg	70.0%	63.4%	75.5%	75.9%	64.3%
Vomiting since surgery	11.5%	14.8%	14.5%	6.9%	10.2%
Pain affecting the ability to cough or to take a deep breath	4.5%	4.3%	2.8%	3.9%	3.1%
Tired since surgery	48.7%	49.5%	32.0%	50.5%	42.0%
Felt vertiginous since surgery	20.1%	20.4%	17.6%	24.5%	20.1%
Pain affecting the mood	20.4%	21.5%	19.2%	19.7%	17.3%
Felt nauseous since surgery	22.4%	26.8%	23.4%	14.7%	19.2%

## Discussion

The aim of our study was to compare general, spinal and combined general and regional anesthesia regarding need for pain medication, patient-reported functional parameters as well as pain scores and patient’s satisfaction in primary knee arthroplasty.

In the past years, many studies have investigated end-points such as cardiovascular and pulmonary complications, length of stay^3^, site infections^9^ and mortality^7^ after both general and regional anesthesia. Furthermore, the postoperative pain management has been put into focus in a large number of studies without taking into account which anesthetic method had been used. To date, not many large-scale studies have compared anesthetic methods with regard to patient’s satisfaction and pain medication needed postoperatively.

In the present study, the use of combined spinal and general, as well as general and peripheral anesthesia was associated with less minimum, maximum and activity pain and improved patients’ postoperative pain management satisfaction on 1^st^ postoperative day.

This differs from the findings of Pope *et al*. who compared 443 patients that underwent knee replacement surgery under different types of anesthesia (general anesthesia, general anesthesia plus femoral nerve block, spinal anesthesia, spinal anesthesia plus femoral nerve block, spinal anesthesia plus intrathecal morphine+/− femoral nerve block)^27^. In this study, postoperative pain at 24 h (VAS-scale) as well as postoperative opioid consumption was documented. In contrast to our NRS results, VAS-scores didn’t differ significantly between the different groups of anesthesiologic technique. Patient satisfaction has not been investigated.

Canakci *et al*. compared spinal anesthesia and psoas compartment with sciatic block (PCS) in 60 patients undergoing total knee arthroplasty^28^. VAS-scores were measured in the postoperative course until 24 h postoperative on a scale from 0–100. In this study no significant difference has been found between the two techniques at 24 h (spinal anesthesia 30 ± 28, PCS 30 ± 10; p = 0.06). Again, direct comparison to our study is not possible as this study investigates two very well-defined techniques and PCS has not been pointed out as an independent category in our study. However, the results are consistent with our findings showing no significant difference in postoperative pain scores between spinal and peripheral anesthesia.

In 2013, Harsten *et al*. published a consecutive and randomized study of 120 patients undergoing total knee arthroplasty under general or spinal anesthesia^6^. Besides, all patients received local infiltration in the perisurgical area towards the end of operation. In this study, postoperative pain was measured taking VAS-scores 0–100 at 0, 2, 4, 6, 10 h postoperatively as well as at 8 a.m. and 2 p.m. both at first and second day after operation at rest, knee flexion, straight knee and walking. As 2 p.m. on first day postop is most likely to be equivalent to our setup of pain evaluation 24 h postop, these are the results we took a closer look at. In all four groups (different movements and rest), the group of general anesthesia had significantly lower pain levels than spinal anesthesia. The results of this study differ strongly from the findings not only of our study but also of many studies of the past years. Nonetheless, because of the randomized setup the results are of high interest. Concerning patient satisfaction, which was also investigated in the study conducted by Harsten *et al*., anesthesia groups did not differ in satisfaction score. Still, many patients receiving spinal anesthesia would have wanted to change anesthesia method if operated again.

Kardash *et al*.^29^ conducted a randomized, double-blinded study investigating 60 patients undergoing total knee replacement under spinal anesthesia receiving either obturator, femoral nerve block or a placebo. The obturator block group didn’t show any difference to spinal-placebo group whereas femoral nerve block reduced pain at rest (p = 0.02) and at movement (p = 0,05) significantly. However, these findings weren’t persistent exceeding 24 hours postop. This may be comparable to our findings because addition of peripheral nerve block to spinal anesthesia only reduced maximum pain levels but didn’t reduce minimum pain levels. In a metaanalysis by Macfarlane *et al*.^30^ regional anesthesia resulted in lower postoperative pain levels.

Although our results are statistically highly significant favoring the combination of general and regional anesthesia with highest NRS differences of 0.9 points for pain, relevance in clinical practice remains unclear. Still, in a large-scale study like the present, the realistic difference of pain on NRS is small.

In knee replacement surgery, the risk of development of chronic pain is reported to be even higher than in e.g. hip replacement surgery. Beswick *et al*. reports chronic pain to occur in 10–34% after total knee replacement and 7–23% after total hip replacement^4^. As the risk of chronic pain seems to be related to high postoperative pain levels^5,6^, improvement of pain management might help to reduce development of chronic pain resulting in faster recovery and mobilization.

The minimally clinically significant NRS difference is a difference of 2, some pain researchers state, although this was only stated for chronic pain^31^. Likewise, our evaluated differences in side effects and subjective well-being were relatively small. This being said, as illustrated by the above mentioned studies, so far no consensus has been found concerning the question which anesthesia technique leads to least postoperative pain.

Furthermore, using combined general and spinal as well as general and peripheral anesthesia was associated with a reduced need for opioid pain medication.

Morphine administration was significantly lower 24 hours postop in the study conducted by Liu *et al*.^32^ in the group of peripheral nerve block in comparison to general anesthesia. This trend persisted after 72 hours. These findings are consistent with Memtsoudis *et al*.^13^ who describes patients receiving a peripheral nerve block (PNB) in total knee arthroplasty requesting significantly less opioids than the ones without (Median of oral morphine equivalents (PNB): 310; (no PNB): 321; p < 0.001). In contrast, Harsten *et al*.^6^ states a significantly lower median morphine consumption 24 hours after surgery in the general anesthesia group (general anesthesia (median) 19 mg, spinal anesthesia (median) 54 mg; p < 0,001). An opioid sparing effect is also described by Szczukowski *et al*.^33^ comparing 40 patients receiving general anesthesia with or without femoral nerve block in a randomized study (GA and FNB 48,1 mg morphine, GA and Placebo 76,2 mg morphine; p = 0,003). In our findings, both combination of general and spinal as well as general and peripheral anesthesia was associated with less opioid consumption than anesthesia techniques effecting only central or peripheral nervous system. Still, low rates of oral morphine consumption can often be explained by higher rates of PCA usage.

Overall, most studies favor peripheral nerve blocks in combination with either spinal or general anesthesia, which is consistent with our findings. Consequentially, least consumption of opioids is due to least amounts of postoperative pain.

Furthermore, combinations of anesthetic techniques (general and spinal, general and peripheral) were associated with a positive influence on some of patients’ subjective parameters such as the ability to move, cough, take a deep breath, tiredness, nausea and impact on mood. Though the magnitude of differences was small, these findings are of high interest because patient’s quality of life after surgery might be ameliorated by functional improvements.

In the past, it was demonstrated that regular benchmarking of pain management programs using the QUIPS questionnaire is able to improve postoperative subjective results^34^.

Limitations to be mentioned include first of all the lack of randomization to anesthetic technique groups. All patients have been treated according to both anesthesiologist’s and surgical experience and possible comorbidities. Thus, the study represents clinical routine and selection bias cannot be excluded even though big data studies reduce this possible bias.

Furthermore, the detailed techniques of the anesthetic type used may differ within the participating departments, which may cause bias; describing these techniques was not the aim of this study.

Because of organizational reasons postoperative pain and pain management have only been recorded on first postoperative day and doses of medication applied could not be homogenized throughout the participating departments. Thus, differences in the further postoperative period could not be pointed out and the intensity of pain medication usage could not be compared regarding e.g. opioid-equivalent doses. Then, groups have been divided to the above-mentioned anesthetic methods, but peripheral anesthesia could not be differentiated into different methods (e.g. femoral or sciatic nerve block); which might be a missing but potentially interesting detail. The use of local infiltration anesthesia (LIA), intensively discussed recently, had not been investigated in this survey. This both might influence NRS value outcome, the latter as groups contain a summarization of patients with and without LIA.

Preoperative use of opioids has not been recorded in this study, another possible limitation. Opioid dependence might influence postoperative pain levels. As the patient quantity is huge in this study, this possible bias is reduced to a minimum as patients with opioid consumption preoperatively occur in all study groups.

Restriction in explanatory power of big data studies is caused by the fact that even small value differences result in significant differences in the final results due to huge data quantity. However, their great importance in evaluation of effectiveness regarding medical interventions in everyday clinical routine remains undeniable.

## Conclusions

Summarizing, the combination of general and spinal as well as general and peripheral anesthesia during primary knee replacement surgery was superior regarding postoperative need for pain medication, side effects, pain scores, patient satisfaction, and subjective well-being parameters in a large number of patients in daily clinical routine. Still, as already mentioned, value differences might be below clinical relevance. Thus, further investigation in terms of large-scale, randomized studies is needed to further analyze the effect of intraoperatively used anesthetic techniques on patient satisfaction and pain scores perioperatively.
